# Nontuberculous mycobacteriosis (*Mycobacterium chelonae*): fatal outcome in a patient with severe systemic lupus erythematosus^☆^

**DOI:** 10.1016/j.abd.2022.12.005

**Published:** 2023-07-03

**Authors:** Bárbara Elias do Carmo Barbosa, Priscila Neri Lacerda, Luana Moraes Campos, Mariângela Esther Alencar Marques, Silvio Alencar Marques, Luciana Patrícia Fernandes Abbade

**Affiliations:** a Department of Infectology, Dermatology, Imaging Diagnosis and Radiotherapy, Faculty of Medicine, Universidade Estadual Paulista, Botucatu, SP, Brazil; b Department of Dermatology, Faculty of Medicine, Universidade Estadual Paulista, Botucatu, SP, Brazil; c Department of Pathology, Faculty of Medicine, Universidade Estadual Paulista, Botucatu, SP, Brazil

Dear Editor,

Patients with systemic lupus erythematosus (SLE) presenting with skin lesions indicate a diagnostic challenge, as they may suggest disease activity, drug eruption, lupus vasculitis, and, more rarely, opportunistic infections.^1^, ^2^ Among the latter, non-tuberculous mycobacteria (NTM) or atypical mycobacteria constitutes an increasing cause of skin infections, especially in immunocompromised patients, including those with autoimmune diseases such as SLE.^3^

This report describes a 37-year-old female patient, who had a previous diagnosis of SLE three years before, on methotrexate 15 mg/week, hydroxychloroquine 400 mg/day, and prednisone 15 mg/day, with no previous history of other immunosuppressive medications. She reported the appearance of skin lesions two months before. Dermatological examination showed erythematous-violaceous macules, papules and nodules (more palpable than visible), indurated, painful, some fistulized and with purulent exudate drainage, distributed on the medial surface of the right thigh (Figure 1, Figure 2).

**Figure 1:**
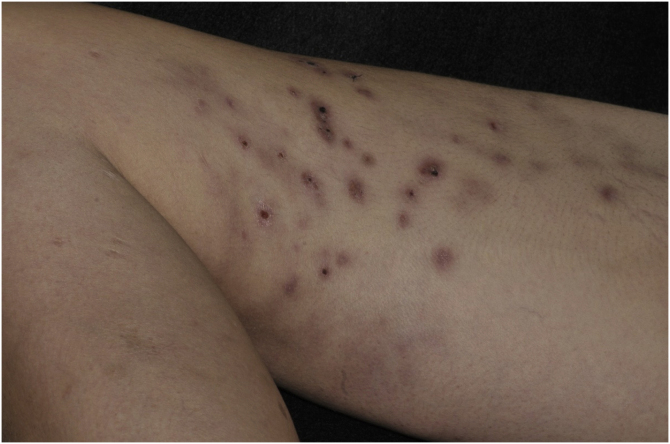
Erythematous-violaceous ulcerated macules and nodules, some covered by hematic crusts, located on the medial surface of the right thigh

**Figure 2:**
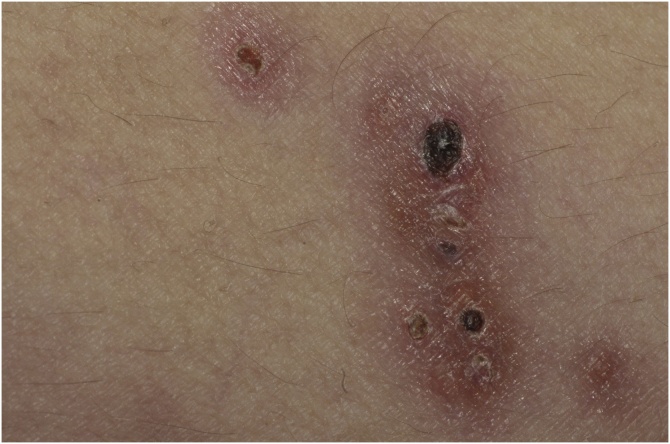
Detail of the erythematous-violaceous ulcerated nodules, with hematic necrotic crusts

She was admitted for investigation of lupus activity and pulmonary thromboembolism (PTE) due to her poor general condition, hematological and respiratory alterations. During hospitalization, she developed fever peaks and was treated with cefepime, imipenem, and vancomycin with no improvement of the skin lesions and the systemic clinical picture.

The skin lesion exudate was sent for a culture of bacteria, fungi and mycobacteria and blood cultures were performed. All results were negative. Serologies for HIV, hepatitis B, hepatitis C and syphilis were non-reactive and bacilloscopy was negative.

A skin biopsy was performed and histopathology showed an epithelioid granulomatous inflammatory process surrounding a cystic necrotic cavity, containing neutrophils (Fig. 3). Ziehl-Neelsen staining showed acid-fast bacilli (AFB) in the cytoplasm of macrophages in the granulomatous process, suggesting nontuberculous mycobacteriosis (Fig. 4). Empirical treatment with rifampicin, ethambutol and azithromycin was initiated, but it was used for only two days, as the patient developed respiratory failure and died due to a thromboembolic event. There was no evidence of systemic mycobacterial infection (negative blood cultures).

**Figure 3:**
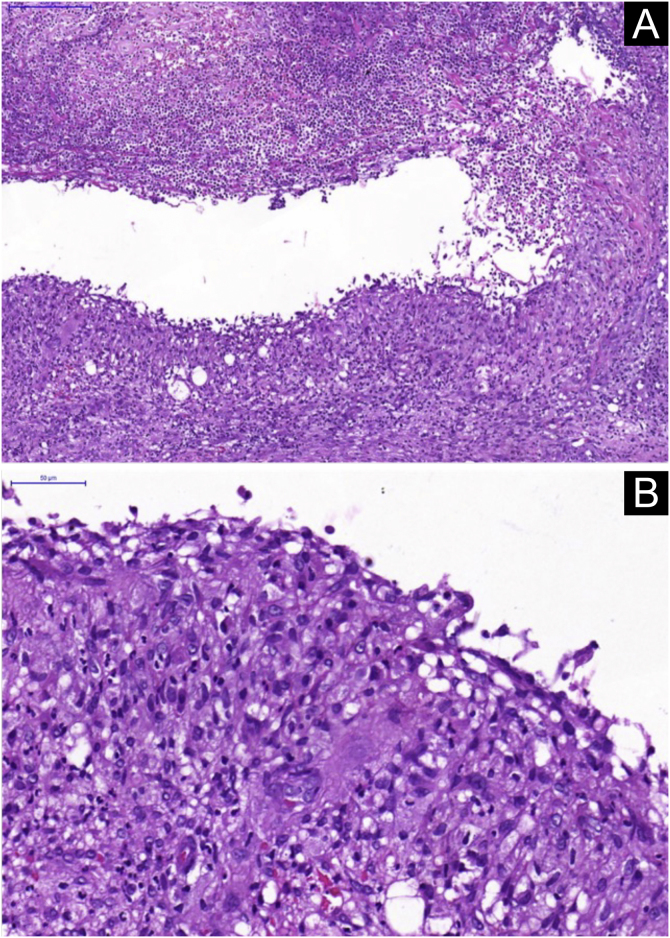
Histopathological analysis. (A) Neutrophilic granulomatous inflammatory process, with superficial and deep involvement of the dermis (Hematoxylin & eosin, ×200). (B) Epithelioid granulomatous inflammatory process surrounding a cystic necrotic cavity containing neutrophils and cell debris (Hematoxylin & eosin, ×400)

**Figure 4:**
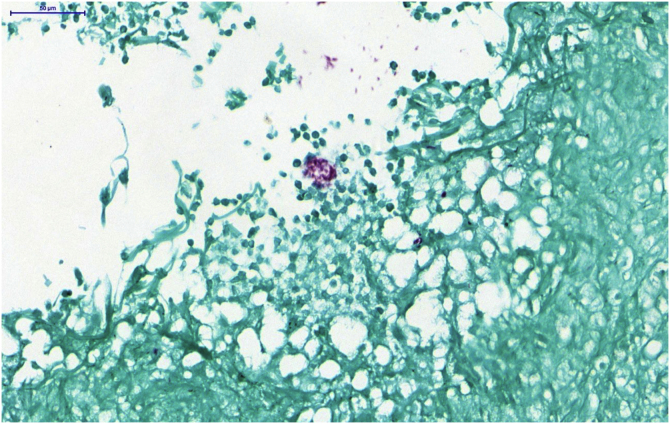
Histopathological analysis: detail showing the presence of acid-fast bacilli (AFB) forming extracellular aggregates (Ziehl Neelsen, ×400)

The biopsy fragment was sent for culture and polymerase chain reaction (PCR) analysis at the Microbiology Laboratory of Instituto Lauro de Souza Lima. The culture showed the presence of fast-growing *Mycobacterium spp.* in five to six days. PCR was performed using the PCR-restriction enzyme analysis (PRA) technique, which consists of amplifying DNA with specific primers for a 441 bp sequence of the hsp65 gene. The PCR product was cleaved with the restriction enzymes *BstE*II and *Hae*III. The result of this analysis was released only after patient death and identified as *Mycobacterium chelonae*.

NTM infections are those caused by pathogenic mycobacteria other than *Mycobacterium tuberculosis* or *M. leprae*, and were formerly known as atypical, anonymous, opportunistic, or unclassified mycobacteriosis.^3^ NTM clinical presentation depends on the species of mycobacteria and can manifest as papules, plaques, nodules, abscesses and ulcers. Histopathological patterns may include non-specific findings of subcutaneous inflammation, abscesses, granulomas, and nodules.^4^, ^5^

The incidence of skin NTM infection has increased in recent decades due to the increased use of immunosuppressive therapy and better detection methods. Histopathological analysis is not species-specific, and some of the species are slow-growing and difficult to culture, making their diagnosis difficult. 5, 6

A high index of suspicion for NTM infections is required in patients with SLE since the initial presentation can mimic several skin manifestations of lupus. The hypothesis should be considered in any patient with indolent skin lesions, especially if routine bacterial cultures are negative. Molecular investigation through polymerase chain reaction (PCR) can increase the sensitivity and specificity, but usually NTM is a late diagnosis and therapy will vary depending on the causative agent.^3^

Almost all species of mycobacteria already identified are capable of causing infection of the skin and subcutaneous tissue, with the main isolated agents being *M. fortuitum*, *M. abscessus*, *M. chelonae*, *M. marinum*, *and M. ulcerans. M. chelonae,* isolated in the present case, is found in aquatic environments, soil, and surgical instruments and is characterized by rapid growth in culture. Localized infections have been reported associated with tattoos, pedicure and cosmetic procedures. Disseminated infection usually occurs in immunocompromised individuals, predominantly affecting the lower extremities.^7^, ^8^

The best therapeutic options described for *M. chelonae* are tobramycin, imipenem, clarithromycin, linezolid and cotrimoxazole. Regardless of the choice of antibiotics, therapy can last for months to over a year. Surgical intervention may also be employed.^3^

A fatal outcome associated with a thromboembolic phenomenon shortly after starting multidrug therapy may be a consequence of a severe infection itself, related to SLE, as well as an adverse event caused by newly introduced drugs, especially rifampicin. Rifampicin-induced coagulopathy is a rare complication, but its influence cannot be ruled out in the present report.^9^ It is noteworthy that the patient had a previous episode of pulmonary thromboembolism but with negative anticardiolipin, IgM and IgG lupus anticoagulant autoantibodies.

This case report aimed to highlight the need to suspect the diagnosis of infections caused by NTM, as opportunistic infections are on the rise in our country. It is also important to warn about the difficulty related to the culture of these microorganisms and the high morbidity and mortality of this disease.

## Financial support

None declared.

## Authors’ contributions

Bárbara Elias do Carmo Barbosa: Drafting and editing of the manuscript; effective participation in propaedeutics; literature review; critical review of the manuscript; approval of the manuscript.

Priscila Neri Lacerda: Drafting and editing of the manuscript; effective participation in propaedeutics; literature review; critical review of the manuscript; approval of the manuscript.

Luana Moraes Campos: Drafting and editing of the manuscript; effective participation in propaedeutics; literature review; critical review of the manuscript; approval of the manuscript.

Mariângela Esther Alencar Marques: Drafting and editing of the manuscript; effective participation in research orientation; effective participation in propaedeutics; literature review; critical review of the manuscript; approval of the manuscript.

Silvio Alencar Marques: Drafting and editing of the manuscript; effective participation in research orientation; effective participation in propaedeutics; literature review; critical review of the manuscript; approval of the manuscript.

Luciana Patrícia Fernandes Abbade: Drafting and editing of the manuscript; effective participation in research orientation; effective participation in propaedeutics; literature review; critical review of the manuscript; approval of the manuscript.

## Conflicts of interest

None declared.

## References

[1] Lenormand C., Lipsker D. (2021). Lupus erythematosus: Significance of dermatologic findings. Ann Dermatol Venereol.

[2] Singh B.K., Singh S. (2020). Systemic lupus erythematosus and infections. Reumatismo.

[3] Nogueira L.B., Garcia C.N., Costa M.S.C.D., Moraes M.B., Kurizky P.S., Gomes C.M. (2021). Non-tuberculous cutaneous mycobacterioses. An Bras Dermatol.

[4] Abbas O., Marrouch N., Kattar M.M., Zeynoun S., Kibbi A.G., Rached R.A. (2011). Cutaneous non- tuberculous Mycobacterial infections: a clinical and histopathological study of 17 cases from Lebanon. J Eur Acad Dermatol Venereol.

[5] Chung J., Ince D., Ford B.A., Wanat K.A. (2018). Cutaneous infections due to nontuberculosis mycobacterium: recognition and management. Am J Clin Dermatol.

[6] Dodiuk-Gad R., Dyachenko P., Ziv M., Shani-Adir A., Oren Y., Mendelovici S. (2007). Nontuberculous mycobacterial infections of the skin: a retrospective study of 25 cases. J Am Acad Dermatol.

[7] Lage R., Biccigo D.G.Z., Santos F.B.C., Chimara E., Pereira E.S.P., DaCosta A. (2015). Mycobacterium chelonae cutaneous infection in apatient with mixed connective tissue disease. An Bras Dermatol.

[8] Rasool S., Afifi A., De Lord D. (2019). Case of atypical cutaneous Mycobacterium chelonae infection in patient of systemic lupus erythematosus after cyclophosphamide therapy. BMJ Case Rep.

[9] Tiperneni R., Khalid F., Fichadiya H., Al-Alwan A., Mohan G., Heis F. (2022). Deranged haemostasis: rifampin-induced coagulopathy. Eur J Case Rep Intern Med.

